# Multivariate Time Series Similarity Searching

**DOI:** 10.1155/2014/851017

**Published:** 2014-05-08

**Authors:** Jimin Wang, Yuelong Zhu, Shijin Li, Dingsheng Wan, Pengcheng Zhang

**Affiliations:** College of Computer & Information, Hohai University, Nanjing 210098, China

## Abstract

Multivariate time series (MTS) datasets are very common in various financial, multimedia, and hydrological fields. In this paper, a dimension-combination method is proposed to search similar sequences for MTS. Firstly, the similarity of single-dimension series is calculated; then the overall similarity of the MTS is obtained by synthesizing each of the single-dimension similarity based on weighted BORDA voting method. The dimension-combination method could use the existing similarity searching method. Several experiments, which used the classification accuracy as a measure, were performed on six datasets from the UCI KDD Archive to validate the method. The results show the advantage of the approach compared to the traditional similarity measures, such as Euclidean distance (ED), cynamic time warping (DTW), point distribution (PD), PCA similarity factor (S_PCA_), and extended Frobenius norm (Eros), for MTS datasets in some ways. Our experiments also demonstrate that no measure can fit all datasets, and the proposed measure is a choice for similarity searches.

## 1. Introduction

With the improving requirement of industries for information and the rapid development of the information technology, there are more and more datasets obtained and stored in the form of multidimensional time series, such as hydrology, finance, medicine, and multimedia. In hydrology, water level, flow, evaporation, and precipitation are monitored for hydrological forecasting. In finance, stock price information, which generally includes opening price, average price, trading volume, and closing price, is used to forecast stock market trends. In medicine, electroencephalogram (EEG) from 64 electrodes placed on the scalp is measured to examine the correlation of genetic predisposition to alcoholism [1]. In multimedia, for speech recognition, the Australian sign language (AUSLAN) is gathered from 22 sensors on the hands (gloves) of a native Australian speaker using high-quality position trackers and instrumented gloves [2].

A time series is a series of observations, *x*_*i*_(*t*); [*i* = 1, …, *n*; *t* = 1, …, *m*], made sequentially through time where *i* indexes the measurements made at each time point *t* [3]. It is called a univariate time series when *n* is equal to 1 and a multivariate time series (MTS) when *n* is equal to or greater than 2.

Univariate time series similarity searches have been broadly explored and the research mainly focuses on representation, indexing, and similarity measure [4]. A univariate time series is often regarded as a point in multidimensional space, so one of the goals of time series representation is to reduce the dimensions (i.e., the number of data points) because of the curse of dimensionality. Many approaches are used to extract the pattern, which contains the main characteristics of original time series, to represent the original time series. Piecewise linear representation (PLA) [5, 6], piecewise aggregate approximation (PAA) [7], adaptive piecewise constant approximation (APCA) [8], and so forth use *l* adjacent segments to represent the time series with length *m*  (*m* ≫ *l*). Furthermore, perceptually important points (PIP) [9, 10], critical point model (CMP) [11], and so on reduce the dimensions by preserving the salient points. Another common family of time series representation approaches transform time series into discrete symbols and perform string operations on time series, for example, symbolic aggregate approximation (SAX) [12], shape description alphabet (SDA) [13], and other symbols generated method based on clustering [14, 15]. Representing time series in the transformation is another large family, such as discrete Fourier transform (DFT) [4] and discrete wavelet transform (DWT) [16] which transform the original time series into frequency domain. After transformation, only the first few or the best few coefficients are chosen to represent the original time series [3]. Many of the representation schemes are incorporated with different multidimensional spatial indexing techniques (e.g., k-d tree [17] and r-tree and its variants [18, 19]) to index sequences to improve the query efficiency during similarity searching. Given two time series *S* and *Q* and their representations *PS* and *PQ*, a similarity measure function *D* calculates the distance between the two time series, denoted by *D*(*PQ*, *PS*) to describe the similarity/dissimilarity between *Q* and *S*, such as Euclidean distance (ED) [4] and the other *Lp* norms, dynamic time warping (DTW) [20, 21], longest common subsequence (LCSS) [22], the slope distance [23], and the pattern distance [24].

The multidimensional time series similarity searches study mainly two aspects, the overall matching and match-by-dimension. The overall matching treats the MTS as a whole because of the important correlations of the variables in MTS datasets. Many overall matching similarity measures are based on principal component analysis (PCA). The original time series are represented by the eigenvectors and the eigenvalues after transformation. The distance between the eigenvectors weighted by eigenvalues is used to describe the similarity/dissimilarity, for example, Eros [3], *S*_PCA_ [25], and *S*_PCA_^*λ*^ [26]. Lee and Choi [27] combined PCA with the hidden Markov model (HMM) to propose two methods, PCA + HMM and PCA + HMM + SVM, to find similar MTS. With the principal components such as the input of several HMMs, the similarity is calculated by combining the likelihood of each HMM. Guan et al. [28] proposed a pattern matching method based on point distribution (PD) for multivariate time series. Local important points of a multivariate time series and their distribution are used to construct the pattern vector. The Euclidean distance between the pattern vectors is used to measure the similarity of original time series.

By contrast, match-by-dimension breaks MTS into multiple univariate time series to process separately and then aggregates them to generate the result. Li et al. [29] searched the similarity of each dimensional series and then synthesized the similarity of each series by the traditional BORDA voting method to obtain the overall similarity of the multivariate time series. Compared to the overall matching, match-by-dimension could take advantage of present univariate time series similarity analysis approaches.

In this paper, a new algorithm based on the weighted BORDA voting method for the MTS *k* nearest neighbor (*k*NN) searching is proposed. Each MTS dimension series is considered as a separate univariate time series. Firstly, similarity searching approach is used to search the similarity sequence for each dimension series; then the similar sequences of each dimensional series are synthesized on the weighted BORDA voting method to generate the multivariate similar sequences. Compared to the measure in [29], our proposed method considers the dimension importance and the similarity gap between the similar sequences and generates more accurate similar sequences.

In the next section, we briefly describe the BORDA voting method and some similarity measures widely used.  Section 3 presents the proposed algorithm to search the *k*NN sequences. Datasets and experimental results are demonstrated in Section 4. Finally, we conclude the paper in Section 5.

## 2. Related Work

In this section, we will briefly discuss BORDA voting method, the method in [29], and the DTW, on which our proposed techniques are based. Notations section contains the notations used in this paper.

### 2.1. BORDA Voting Method

BORDA voting, a classical voting method in group decision theory, is proposed by Jena-Charles de BORDA [30]. Supposing *k* is the number of winners, *c* is the number of candidates; *e* electors express their preference from high to low in the sort of candidates. To every elector's vote, the candidate ranked first is provided *e* points (called voting score), the second candidate *e*-1 points, followed by analogy, and the last one is provided 1 point. The accumulated voting score of the candidate is BORDA score. The candidates, BORDA scores in the top *k*, are called BORDA winners.

### 2.2. Similarity Measure on Traditional BORDA Voting

Li et al. [29] proposed a multivariate similarity measure based on BORDA voting, denoted by *S*_BORDA_; the measure is divided into two parts: the first one is the similarity mining of univariate time series and the second one is the integration of the results obtained in the first stage by BORDA voting. In the first stage, a certain similarity measure is used to query *k*NN sequences on univariate series of each dimension in the MTS. In the second stage, the scores of each univariate similar sequence are provided through the rule of BORDA voting. The most similar sequence scores *i* points, the second scores *i*-1, followed by a decreasing order, and the last is 1. The sequences with same time period or very close time period will be found in different univariate time series. According to the election rule, the sequences whose votes are less than the half of dimension are eliminated; then the BORDA voting of the rest of sequences is calculated. If a sequence of some certain time period appears in the results of *p* univariate sequences and its scores are *s*_1_, *s*_2_,…, *s*_*p*_, respectively, then the similarity score of this sequence is the sum of all the scores. In the end, the sequence with the highest score is the most similar to the query sequence.

### 2.3. Dynamic Time Warping Distance

Dynamic programming is the theoretical basis for dynamic time warping (DTW). DTW is a nonlinear planning technique combining time and distance measure, which was firstly introduced to time series mining areas by Berndt and Clifford [20] to measure the similarity of two univariate time series. According to the minimum cost of time warping path, the DTW distance supports time axis stretching but does not meet the requirement of triangle inequality.

## 3. The Proposed Method

In the previous section, we have reviewed the similarity measure on traditional BORDA voting, *S*_BORDA_, for multivariate time series. In this section, we propose a dimension-combination similarity measure based on weighted BORDA voting, called *S*_WBORDA_, for MTS datasets *k*NN searching. The similarity measure can be applied for the whole sequence matching similarity searching and the subsequence matching similarity searching.

### 3.1. S_WBORDA_: Multivariate Similarity Measure on Weighted BORDA Voting

*S*_BORDA_ takes just the order into consideration, without the actual similarity gap between two adjacent similar sequences that may lead to rank failure for the similar sequences. For example, assuming the four candidates *r*_1_, *r*_2_, *r*_3_, *r*_4_ take part in race, the first round position is *r*_1_, *r*_2_, *r*_3_, *r*_4_, the second is *r*_2_, *r*_1_, *r*_4_, *r*_3_, the third is *r*_4_, *r*_3_, *r*_1_, *r*_2_, and the last is *r*_3_, *r*_4_, *r*_2_, *r*_1_. The four runners are all ranked number 1 with traditional BORDA score (10 points), because of considering only the rank order, but without the speed gap of each runner in the race. In our proposed approach, we use the complete information of candidate, including the order and the actual gap to neighbor.

The multivariate data sequences *S* with *n* dimensions are divided into *n* univariate time series, and each dimension is a univariate time series. Given multivariate query sequence *Q*, to search the multivariate *k*NN sequences, each univariate time series is searched separately. For the *j*th dimension time series, the *k*′ + 1 nearest neighbor sequences are *s*_0_, *s*_1_, …, *s*_*k*′_, where *k*′ is equal or greater than *k* and *s*_0_ is the *j*th dimension series of *Q* and is considered to be the most similar to itself. The distances between *s*_1_, …, *s*_*k*′_ and *s*_0_ are *d*_1_, …, *d*_*k*′_, respectively, where *d*_*i*−1_ is less than or equal to *d*_*i*_  and  *d*_*i*_ − *d*_*i*−1_ describes the similarity gap between *s*_*i*_ and *s*_*i*−1_ to *s*_0_. Let the weighted voting score of *s*_0_ be *k*′ + 1 and let *s*_*k*′_ be 1; the weighted voting score of the sequence *s*_*i*_, vs_*i*_, is defined by

(1) $$\begin{array}{c} {\text{vs}}_{i}=w_{j}\left(1+k^{\prime }\left(1-\frac{d_{i}}{d_{k^{\prime }}}\right)\right)\;\left(i=1,\ldots ,k^{\prime }-1\right), \end{array}$$

where *w* is a weight vector based on the eigenvalues of the MTS dataset, ∑_*j*=1_^*n*^ *w*_*j*_ = 1, and *w*_*j*_ represents the importance of the *j*th dimension series among the MTS. vs_*i*_ is inversely proportional to *d*_*i*_; that is, *s*_0_ is the baseline; the higher similarity gap between *s*_*i*_ and *s*_0_ is, the lower weighted BORDA score *s*_*i*_ will get.

**Figure 1:**
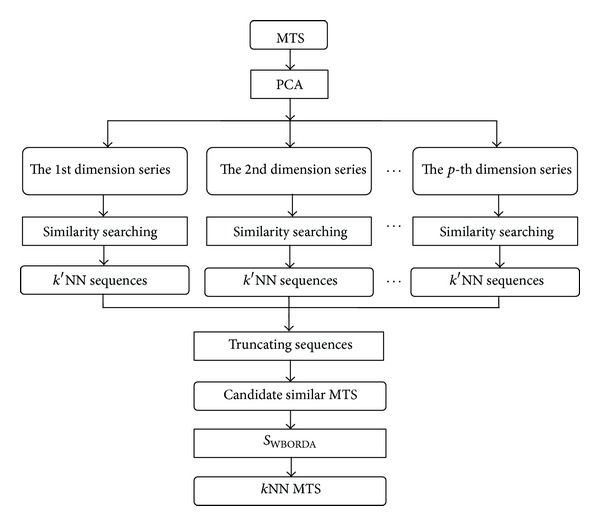
The model of similarity searching on weighted BORDA voting.

We accumulate the weighted voting score of each item in a candidate multivariate similar sequence and then obtain its weighted BORDA score. The candidate sequences are ranked on weighted BORDA scores, and the top *k* are the final similar sequences to *Q*. The model of similarity searching based on weighted BORDA voting is shown in Figure 1.

In the model of Figure 1, firstly, PCA is applied on original MTS and transforms it to new dataset *Y* whose variables are uncorrelated with each other. The first *p* dimensions series which contain most of characteristics of the original MTS are retained to reduce dimensions. Furthermore, univariate time series similarity searching is performed to each dimension series in *Y* and finds out the univariate *k*′NN sequences; *k*′ should be equal or greater than the final *k*. Moreover, *k*′NN sequences are truncated to obtain the candidate multivariate similar sequences. Finally, *S*_WBORDA_ is performed on candidate multivariate similar sequences to obtain the *k*NN of query sequences. Intuitively, *S*_WBORDA_ measures the similarity from different aspects (dimensions) and synthesizes them. The more aspects (dimensions) from measured sequences is similar to the query sequences, the more similar the sequence is to the query sequences of the whole. The following sections describe the similarity searching in detail.

### 3.2. Performing PCA on Original MTS

In our proposed method, all MTS dimension series are considered independent of each other, but, in fact, correlation exists among them more or less, so PCA is applied to the MTS which can be represented as a matrix *X*_*m*×*n*_ and *m* represents the length of series, and *n* is the number of dimensions (variables). Each row of *X* can be considered as a point in *n*-dimensional space. Intuitively, PCA transforms dataset *X* by rotating the original *n*-dimensional axes and generating a new set of axes. The principal components are the projected coordinate of *X* on the new axes [3].

Performing PCA on a multivariate dataset *X*_*m*×*n*_ is based on the correlation matrix or covariance matrix of *X* and results in two matrices, the eigenvectors matrix *C*_*n*×*n*_ and the variances matrix *L*_*n*×1_. Each column of *C*_*n*×*n*_, called eigenvector, is a unit vector, geometrically, and it presents the new axes position in the original *n*-dimensional space. The variances matrix element *L*_*i*×1_, called eigenvalue, provides the variance of the *i*th principal component. The matrix of the new projected coordinates *D*_*m*×*n*_ of the original data can be calculated by *D* = *X* · *C*. The first dimension univariate time series of *D* is the first principal component and accounts for the largest part of variances presented in the original *X*; the *i*th principal component accounts for the largest part of the remaining variances and is orthogonal to the 1st, 2nd,…, and *i* − 1th dimensions. Select the first *p* components *D*_*m*×*p*_, which retain more than, for example, 90% of the total variation presented in the original data representing *X*. Thus, the dimensionality reduction may be achieved, as long as *p* ≪ *n*. Geometrically, the original *X* is projected on the new *p*-dimensional space.

In whole sequence matching similarity searching, we apply PCA to all MTS items and retain *p* components so that more than, for example, 90% of the total variations are retained in all MTS items at least.

### 3.3. Truncating Univariate Similar Sequences

In candidate similar MTS, each dimension series starts at the same time. However, the similar sequences of each dimension time series may not start at the same time. The similar sequences with close start time of each dimension could be treated as in the same candidate similar MTS and truncated. The truncation includes four steps: grouping the sequences, deleting the isolated sequences, aligning the overlapping sequences, and reordering the sequences. After truncation, the candidate multivariate similar sequences could be obtained.

**Figure 2:**
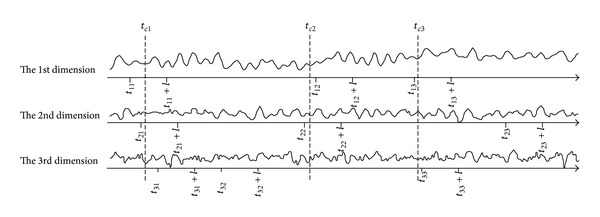
Truncating similar sequences for subsequence matching.

The truncation for whole sequence matching similarity searching is just a special case of subsequence matching, so we introduce the truncation for subsequence matching. In Figure 2, 3NN sequences are searched for multivariate query sequences with length *l*, and the application of PCA on the data MTS results in the principal component series with three dimensions. 3NN searching is performed on each dimension principal component series. The 3NN sequences of first dimension are *s*_11_ (the subsequence from *t*_11_ to *t*_11_ + *l*), *s*_12_ (from *t*_12_ to *t*_12_ + *l*), and *s*_13_ (from *t*_13_ to *t*_13_ + *l*). The univariate similar sequences are presented according to their occurrence time, and the present order does not reflect the similarity order to the query sequence. The 3NN sequences of the second dimension are *s*_21_ (from *t*_21_ to *t*_21_ + *l*), *s*_22_ (from *t*_22_ to *t*_22_ + *l*), and *s*_23_ (from *t*_23_ to *t*_23_ + *l*), and these of the third dimension are *s*_31_ (from *t*_31_ to *t*_31_ + *l*), *s*_32_ (from *t*_32_ to *t*_32_ + *l*), and *s*_33_ (from *t*_33_ to *t*_33_ + *l*).

*(1) Grouping the Univariate Similar Sequences*. The univariate similar sequences of all dimensions are divided into groups, so that in each group, for any sequence *s*, at least one sequence *w*, which overlaps with *s* over the half length of sequence *l*, could be found. The univariate similar sequence, which does not overlap with any other similar sequences, will be put into a single group just including itself. In Figure 2, all the similar sequences are divided into five groups. The group g1 includes *s*_11_, *s*_21_, *s*_31_. *s*_11_, *s*_21_ overlaps with *s*_21_, *s*_31_, respectively, and the overlapping lengths are all over half of the length *l*, group g2 includes *s*_32_, group g3 includes *s*_12_, *s*_22_, group g4 includes *s*_13_, *s*_33_, and group g5 includes *s*_23_. 

*(2) Deleting the Isolated Sequences*. The group, in which the number of similar sequences is less than half number of the dimensions, is called an isolated group, and the similar sequences in isolated group are called isolated similar sequences. In Figure 2, the number of similar sequences in group g2 or g5 is less than half of the number of dimensions, that is, 3, so the similar sequences in them are deleted. 

*(3) Aligning the Overlapping Sequences*. The sequences in the same group are aligned to generate the candidate multivariate similar sequences. For one group, the average start time *t* of all the included sequences is calculated; then the subsequence from *t* to *t* + *l*, denoted by cs, is the candidate multivariate similar sequence. Each dimension series of cs is regarded as the univariate similar sequences. The similarity distance between cs and query sequence is recalculated by the selected univariate similarity measure dimension by dimension; if the group contains the *i*th dimension similar sequence, then the corresponding similarity distance is set to the *i*th dimension series of cs to reduce computation. In Figure 2, for group g1, the average of *t*_11_, *t*_21_, *t*_31_ *t*_*c*1_ is calculated; then the subsequence *s*_*tc*1_, from *t*_*c*1_ to *t*_*c*1_ + *l*, is the candidate multivariate similar sequence. For group g3, the similarity distance between the 2nd dimension series of *s*_*tc*2_ and the query sequence should be recalculated. The same alignment operation is performed on group g4 to obtain the candidate multivariate sequence *s*_*tc*3_. 

*(4) Reordering the Candidate Similar Sequences*. For each dimension, the candidate univariate similar sequences are reordered by the similarity distance calculated in Step (3). After reordering, *S*_WBORDA_ is used to synthesize the candidate similar sequences and generate the multivariate *k*NN sequences.

In whole matching *k*NN searching, the similar sequences are either whole overlapping or not overlapping each other at all, and the truncation steps are the same as those of the subsequence matching.

### 3.4. Computing Weights

By applying PCA to the MTS, the principal components series and the eigenvalues, which can represent the variances for principal components, are obtained. When we calculate the weighted BORDA score, we take into consideration both the similarity gap and the dimension importance for *S*_WBORDA_. The heuristics proposed algorithm in [3] is used to calculate the weight vector *w* based on the eigenvalues. Variances are aggregated by a certain strategy, for example, min, mean, and max, on eigenvalues vectors dimension by dimension, and the vector *w*〈*w*_1_, *w*_2_, …, *w*_*k*_〉 is obtained. The weight vector element is defined by

(2) $$\begin{array}{c} w_{i}=\frac{f\left(V_{i}\right)}{\sum_{j=1}^{k}f\left(V_{j}\right)\;}\;\;i=1,\ldots ,p, \end{array}$$

where *f*() denotes the aggregating strategy. *V*_*i*_ means the variance vector of *i*th dimension of MTS items. Generally, for subsequence matching, *V*_*i*_ includes one element, and whole matching is greater than 1. Intuitively, each *w*_*i*_ in the weight vector represents the aggregated variance for all the *i*th principal components. The original variance vector could be normalized before aggregation.

## 4. Experimental Evaluation

In order to evaluate the performance of our proposed techniques, we performed experiments on six real-world datasets. In this section, we first describe the datasets used in the experiments and the experiments methods followed by the results.

### 4.1. Datasets

The experiments have been conducted on four UCI datasets, [31] electroencephalogram (EEG), Australian sign language (AUSLAN), Japanese vowel (JV), and robot execution failure (REF), which are all labeled MTS datasets.

The EEG contains measurements from 64 electrodes placed on the scalp and sampled at 256 Hz to examine EEG correlates of genetic predisposition to alcoholism. Three versions, the small, the large, and the full, are included in this dataset according to the volume of the original data. We utilized the large dataset containing 600 samples and 2 classes.

The AUSLAN2 consists of samples of AUSLAN (Australian sign language) signs. 27 examples of each of 95 AUSLAN signs were captured from 22 sensors placed on the hands (gloves) of a native signer. In total, there are 2565 signs in the dataset.

The JV contains 640 time series of 12 LPC cepstrum coefficients taken from nine male speakers. The length of each time series is in the range 7–29. It describes the uttering of Japanese vowels /ae/ by a speaker successively. The dataset contains two parts: training and test data; we utilized the training data which contains 270 time series.

The REF contains force and torque measurements on a robot after failure detection. Each failure is characterized by 6 forces/torques and 15 force/torque samples. The dataset contains five subdatasets LP1, LP2, LP3, LP4, and LP5; each of them defines a different learning problem. The LP1, LP4, and LP5 subdatasets were utilized in the experiment. LP1 which defines the failures in approach to grasp position contains 88 instances and 4 classes, LP4 contains 117 instances and 3 classes, and LP5 contains 164 instances and 5 classes. A summary is shown in Table 1.

### 4.2. Method

In order to validate our proposed similarity measure *S*_WBORDA_, 1NN classification, and 10-fold cross validation are performed [32]. That is, each dataset is divided into ten subsets, 1-fold for testing and the rest 9 for training. For each query item in the testing set, 1NN is searched in the training set and the query item is classified according to the label of the 1NNs, and the average precision is computed across all the testing items. The experiment is repeated 10 times for different testing set and training set, and 10 different error rates are obtained; then the average error across all 10 trials is computed for 10-fold cross validation. We performed 10 times 10-fold cross validation and computed the average error across all 10-fold cross validations to estimate the classification error rate. The similarity measures tested on our experiments include *S*_BORDA_, PD, Eros, and *S*_PCA_. DTW is selected as the univariate similarity measure for *S*_BORDA_ and *S*_WBORDA_. They are denoted as *S*_BORDA_DTW_ and *S*_WBORDA_DTW_, respectively. For DTW, the maximum amount of warping *Q* is decreased to 5% of the length. DTW has been extensively employed in various applications and time series similarity searching, because DTW can be applied to two MTS items warped in the time axis and with different lengths.

All other measures except PD and Eros require determining the number of components *p* to be retained. Classification has been conducted for consecutive values of *p* which retain more than 90% of total variation, until the error rate reaches the minimum. The number which retains less than 90% of total variation is not considered. For Eros, the experiments are conducted as proposed in [3]. The Wilcoxon signed-rank test is used to ascertain if *S*_BORDA_DTW_ yields an improved classification performance on multiple data sets in general. PCA is performed on the covariance matrices of MTS datasets.

### 4.3. Results

The classification error rates are presented in Table 2 in the form of percentages. Although experiments have been conducted for various *p*, that is, the number of principal components (for *S*_BORDA_DTW_, *S*_WBORDA_DTW_, and *S*_PCA_), only the best classification accuracies are presented.

Firstly, we will compare similarity measures with respect to each dataset, respectively. For the EEG dataset, *S*_PCA_ produces the best performance and performs significantly better than the others. With regard to the AUSLAN2 dataset, Eros produces the lowest classification error rate and PD gives very poor performance. For the overall matching method, for example, Eros, *S*_PCA_ performs better than the others. For the JV dataset, *S*_PCA_ gives the best performance and the *S*_BORDA_DTW_ makes the poorest performance. For the LP1 dataset, *S*_WBORDA_DTW_ makes the best performance. For the LP4 dataset, *S*_WBORDA_DTW_ makes the best performance and *S*_BORDA_DTW_ and *S*_WBORDA_DTW_ perform better than others. In the end, for the LP5 dataset, *S*_WBORDA_DTW_ gives the best performance.

Finally, the similarity measures are compared for all the datasets. Between *S*_BORDA_DTW_ and *S*_WBORDA_DTW_, the Wilcoxon signed-rank test reports that *P* value equals 0.043 (double side) and shows that the algorithms are significantly different. With 5% significance level, *S*_WBORDA_DTW_ has made better performance over *S*_BORDA_DTW_. Compared to Eros and *S*_PCA_, the *S*_WBORDA_DTW_ has better performance on LP1, LP4, and LP5. But it shows poor performance on EEG, AULSAN2, and JV.  Table 3 shows the number of principal components, which just retain more than 90% of total variation, in experiment datasets. For LP1, LP4, and LP5, the first few principal components retained most of the variation after PCA performing, but for EEG, AUSLAN2, and JV, to retain more than 90% of total variation, more principal components should be retained. *S*_WBORDA_DTW_ searches the similar sequences dimension by dimension and then synthesizes them; it is hard to generate the aligned candidate multivariate similar sequences when many principal components are contained in the principal component series. Furthermore, for the datasets, for example, EEG, AUSLAN2, and JV, the first few principal components could not retain sufficient information of original series, and *S*_WBORDA_DTW_ produces poor precision. *S*_WBORDA_DTW_ could make better performance on the datasets which aggregate the most variation in the first few principal components after PCA.

## 5. Conclusion and Future Work

A match-by-dimension similarity measure for MTS datasets, *S*_WBORDA_, is proposed in this paper. This measure is based on principal component analysis, weighted BORDA voting method, and univariate time series similarity measure. In order to compute the similarity between two MTS items, *S*_WBORDA_ performs PCA on the MTS and retains *k* dimensions principal component series, which present more than 90% of the total variances. Then a univariate similar analysis is applied to each dimension series, and the univariate similar sequences are truncated to generate candidate multivariate similar sequences. At last, the candidate sequences are ordered by weighted BORDA score, and the *k*NN sequences are obtained. Experiments demonstrate that our proposed approach is suitable for small datasets. The experimental result has also shown that the proposed method is sensitive to the number of the classes in datasets. In the further work, it will be investigated furtherly.

In the literature, at present, there are still not so many studies in similarity analysis for MTS. In the future, we will explore new integration methods.

## Figures and Tables

**Table 1:** Summary of datasets used in the experiments.

Dataset	Number of variables	Mean length	Number of instances	Number of classes
EEG	64	256	600	2
AUSLAN2	22	90	2565	95
JV	12	16	270	9
LP1	6	15	88	4
LP4	6	15	117	3
LP5	6	15	164	5

**Table 2:** Classification error rate (%).

	EEG	AUSLAN2	JV	LP1	LP4	LP5
S_BORDA_DTW_	_(14)_27.7	_(4)_52.6	_(5)_53.4	_(3)_15.9	_(3)_9.8	_(4)_24.0
S_WBORDA_DTW_	_(14,max)_27.7	_(4,max)_50.4	_(6,max)_52.4	_(4,mean)_13.0	_(3,mean)_8.1	_(3,mean)_21.3
PD	38.4	68.9	45.2	14.1	12.3	41.2
Eros	_(max)_5.8	_(mean)_11.1	_(mean)_30.6	_(max)_20.6	_(mean)_10.3	_(mean)_35.5
S_PCA_	_(14)_1.2	_(4)_13.2	_(12)_29.5	_(3)_24.7	_(4)_20.0	_(3)_35.2

(Numbers in parentheses indicate the *p*, i.e., the number of principal components retained, “max” and “mean” indicate the aggregating functions for weight *w.*)

**Table 3:** Contribution of principal components.

	EEG	AUSLAN2	JV	LP1	LP4	LP5
Number of variables	64	22	12	6	6	6
Number of retained principal components*	14	4	5	3	3	3
Retained variation (%)	90.1	92.7	93.9	92.4	94.8	91.2

(*For every dataset, if the number of principal components is less than the number in Table 3, the retained variation will be less than 90%.)

## Notations

*n*: The number of MTS dimensions
*m*: The number of observations in a MTS
*p*: The number of retained principal components
*k*: The number of multivariate nearest neighbor sequences
*k*′: The number of univariate nearest neighbor sequences, usually greater than or equal *k*
*s*_*i*_: The *i*th univariate similar sequence, 1 ≤ *i* ≤ *k*′
*d*_*i*_: The distances between *s*_*i*_ and *s*_0_, 1 ≤ *i* ≤ *k*′
vs_*i*_: Weighted voting score of the *i*th univariate similar sequence, 1 ≤ *i* ≤ *k*′
*C*_*n*×*n*_: Eigenvectors matrix of PCA
*L*_*n*×1_: Variances matrix of PCA
*f*(): The aggregating strategy of variances
*w*: Weight vector of principal components series
*V*_*i*_: The *i*th dimension variances vector of MTS items, 1 ≤ *i* ≤ *p*.
